# High Bleeding Risk Patients Treated with Very Thin-Strut Biodegradable Polymer or Thin-Strut Durable Polymer Drug-Eluting Stents in the BIO-RESORT Trial

**DOI:** 10.1007/s10557-018-6823-9

**Published:** 2018-08-24

**Authors:** Paolo Zocca, Marlies M. Kok, Liefke C. van der Heijden, Peter W. Danse, Carl E. Schotborgh, Martijn Scholte, Marc Hartmann, Gerard C. M. Linssen, Carine J. M. Doggen, Clemens von Birgelen

**Affiliations:** 1 Department of Cardiology, Thoraxcentrum Twente, Medisch Spectrum Twente, Postbus 50.000, 7500 KA Enschede, the Netherlands; 2 Department of Cardiology, Rijnstate Hospital, Arnhem, the Netherlands; 3 Department of Cardiology, Haga Hospital, Den Haag, the Netherlands; 4 Department of Cardiology, Albert Schweitzer Hospital, Dordrecht, the Netherlands; 5 Department of Cardiology, Ziekenhuisgroep Twente, Almelo and Hengelo, the Netherlands; 6 Department of Health Technology and Services Research, Faculty of Behavioural, Management and Social Sciences, Technical Medical Centre, University of Twente, Enschede, the Netherlands

**Keywords:** Biodegradable polymer, Drug-eluting stent, Durable polymer, High bleeding risk, Percutaneous coronary intervention

## Abstract

**Purpose:**

Patients with high bleeding risk (HBR) who undergo percutaneous coronary intervention also have an increased risk of ischemic events and represent an overall high-risk population. The coating of durable polymer drug-eluting stents (DP-DES) may induce inflammation and delay arterial healing, which might be reduced by novel biodegradable polymer DES (BP-DES). We aimed to evaluate the safety and efficacy of treating HBR patients with very thin-strut BP-DES versus thin-strut DP-DES.

**Methods:**

Participants in BIO-RESORT (NCT01674803), an investigator-initiated multicenter, randomized all-comers trial, were treated with very thin-strut BP-DES (Synergy or Orsiro) or thin-strut DP-DES (Resolute Integrity). For the present analysis, patients were classified following HBR criteria based on previous trials. The primary endpoint was target vessel failure: a composite of cardiac death, target vessel-related myocardial infarction, or target vessel revascularization at 1 year.

**Results:**

Of all 3514 patients, 1009 (28.7%) had HBR. HBR patients were older (*p* < 0.001) and had more co-morbidities than non-HBR patients (*p* < 0.001). At 1-year follow-up, HBR patients had significantly higher rates of target vessel failure (6.7 vs. 4.2%, *p* = 0.003), cardiac death (1.9 vs. 0.4%, *p* < 0.001), and major bleeding (3.3 vs. 1.5%, *p* = 0.001). Of all 1009 HBR patients, 673 (66.7%) received BP-DES and 336 (33.3%) had DP-DES. The primary endpoint was met by 43/673 (6.5%) patients treated with BP-DES and 24/336 (7.3%) treated with DP-DES (HR 0.88 [95%CI 0.54–1.46], *p* = 0.63). There were no significant between-group differences in the most global patient-oriented clinical endpoint (9.7 vs. 10.5%, HR 0.92 [95%CI 0.61–1.39], *p* = 0.69) and other secondary endpoints.

**Conclusions:**

At 1-year follow-up, very thin-strut BP-DES showed similar safety and efficacy for treating HBR patients as thin-strut DP-DES.

**Electronic supplementary material:**

The online version of this article (10.1007/s10557-018-6823-9) contains supplementary material, which is available to authorized users.

## Introduction

Most patients undergoing percutaneous coronary intervention (PCI) are currently treated with drug-eluting stents (DES) that elute an antiproliferative drug from their coating [1, 2]. But the life-long presence of durable polymers (DP) on the metallic stent struts may induce vessel wall inflammation and delay arterial healing, which can result in adverse clinical events such as stent thrombosis or myocardial infarction (MI) [3].

Novel very thin-strut biodegradable polymer DES (BP-DES) were designed to overcome these limitations of DP-DES and merely leave a bare metal stent in place after the polymer has been absorbed [1, 2]. International guidelines recommend the use of contemporary DES over first-generation DES and bare metal stents in all patients undergoing PCI [4]. There is no preference for newer-generation BP-DES over DP-DES, as both DES groups have shown to improve clinical outcome as compared to first-generation DES and bare metal stents. Despite the theoretical advantage of BP-DES, meta-analyses of clinical trials showed no unequivocal benefit of BP-DES over contemporary DP-DES [5–8], but there might still be an advantage for BP-DES in high-risk subgroups. Patients who are at high bleeding risk (HBR) may represent such a patient population.

In clinical practice, a substantial proportion of PCI patients are at HBR [9, 10]. They also have an increased risk of *ischemic* events and thus, represent a population with an *overall high risk* of adverse clinical outcome [11]. Comparative studies of HBR patients treated with BP-DES versus DP-DES are lacking, and potential benefits of BP-DES for treating this high-risk subgroup are undetermined. Therefore, in the present study, we analyzed the clinical outcome data at 1-year follow-up of the large-scale BIO-RESORT randomized all-comer trial [12] to evaluate in HBR patients the safety and efficacy of PCI with very thin-strut BP-DES versus thin-strut DP-DES.

## Materials and Methods

### Study Design and Population

The present study is an explorative analysis of the BIO-RESORT trial (Comparison of Biodegradable Polymer and Durable Polymer Drug-Eluting Stents in an All-Comers Population) [TWENTE III]) [12]. The study design and population, as well as the main 1-year results have been published previously [12, 13]. In brief, BIO-RESORT (ClinicalTrials.gov: NCT01674803) is an investigator-initiated, multicenter, randomized controlled trial in 3514 all-comers who were treated with PCI. The study was performed at four clinical sites in the Netherlands (Thoraxcentrum Twente, Medisch Spectrum Twente, Enschede; Rijnstate Hospital, Arnhem; Haga Hospital, The Hague; Albert Schweitzer Hospital, Dordrecht). Patients were eligible if they were 18 years or older, capable of providing informed consent, and required a PCI with DES implantation according to clinical guidelines or the operators’ judgment. All coronary syndromes were permitted and there was no limit for the number of lesions to be treated or for lesion characteristics. Only few exclusion criteria were applied [13]. Web-based computer-generated allocation sequences randomly assigned study participants in a 1:1:1 fashion to treatment with a very thin-strut, biodegradable polymer everolimus-eluting stent (Synergy; Boston Scientific, Natick, MA, USA), a very thin-strut, biodegradable polymer sirolimus-eluting stent (Orsiro; Biotronik AG, Bülach, Switzerland), or a thin-strut, durable polymer zotarolimus-eluting stent (Resolute Integrity; Medtronic, Santa Rosa, CA, USA). For the present analysis, HBR patients treated with BP-DES (i.e., Synergy and Orsiro) were compared to patients treated with DP-DES (i.e., Resolute Integrity). Dual antiplatelet therapy was generally prescribed for 6–12 months, according to international and local guidelines. The only exception was patients on oral anticoagulation therapy, in whom aspirin was usually discontinued after 1–6 months, while clopidogrel was often prescribed for 1 year. The trial complied with the CONSORT 2010 Statement and Declaration of Helsinki and was approved by the Medical Ethics Committee Twente and the institutional review boards of all participating centers. All trial participants provided written informed consent.

As there is no generally accepted definition of HBR for patients with coronary artery disease who undergo PCI, we applied the vast majority of HBR criteria applied in previous HBR studies [10, 14, 15], using data available in the BIO-RESORT study database. Patients in the present analysis were classified as HBR if they fulfilled at least one of the following criteria: (1) age ≥ 75 years; (2) current use of oral anticoagulation therapy; (3) hemoglobin < 11 g/dl; (4) platelet count < 100,000/mm^3^; (5) pevious gastro-intestinal bleeding within last 12 months before the index procedure; (6) previous stroke within 12 months before the index procedure; (7) previous intracranial bleeding; (8) severe renal insufficiency requiring dialysis; (9) current use of non-steroidal anti-inflammatory drugs. As (10) planned major surgery in the next 6 months after the index PCI had been an exclusion criterion of the BIO-RESORT trial, none of the BIO-RESORT patients fulfilled this criterion. In contrast to the previous HBR trials, we did not have information of previously diagnosed malignancy and severe liver disease (e.g., cirrhosis) available in our database, and therefore, we might have missed some of these HBR patients. However, if patients with severe liver disease had reduced levels of hemoglobin or platelet count, they anyway were classified as HBR.

### Follow-Up

One-year clinical follow-up data were obtained at visits to outpatient clinics or, if not feasible, by telephone or medical questionnaire. Trial coordination and data management were performed by the clinical research organization Cardiovascular Research and Education (CRE, Enschede, the Netherlands). Data monitoring and independent clinical event adjudication were performed by an independent clinical research organization (Diagram, Zwolle, the Netherlands).

### Definitions of Clinical Endpoints

All clinical endpoints were pre-specified and defined according to the Academic Research Consortium (ARC) criteria [16, 17]. The primary endpoint of this study was the 1-year rate of target vessel failure (TVF), a composite of cardiac death, target vessel-related MI, or clinically indicated target vessel revascularization. Death was considered as cardiac unless an unequivocal non-cardiac cause could be established. Secondary endpoints included any death, any MI, target lesion failure (TLF, a composite of cardiac death, target lesion-related MI, or clinically indicated target lesion revascularization), major adverse cardiac events (MACE, a composite of any death, any MI, emergent coronary bypass revascularization, or clinically indicated target lesion revascularization), the most global patient-oriented composite endpoint (POCE, a composite of any death, any MI, or any revascularization), stent thrombosis, and major bleeding. The latter was defined as any Bleeding Academic Research Consortium (BARC) type 3 or 5 and/or Thrombolysis in Myocardial Infarction (TIMI) major bleeding (i.e., including coronary artery bypass grafting-related major bleeding) [18, 19].

### Statistical Analysis

Continuous data are reported as mean ± standard deviation and categorical data as numbers and percentages. Student’s *t* test and Chi-square test (or Fisher’s exact test, as appropriate) were used to compare differences in baseline characteristics. Time to clinical endpoints was calculated using Kaplan-Meier analyses, and the log-rank test was applied for between-group comparisons. Hazard ratios were computed using Cox proportional hazard regression analyses. We used logistic regression for interaction between subgroups and treatment with regard to the primary endpoint in analogy with the BIO-RESORT trial [12]. All *p* values are two-sided and considered significant if being < 0.05. Data analysis was performed with SPSS, Version 22.0 (IBM Corp., Armonk, NY, USA).

## Results

**Fig. 1:**
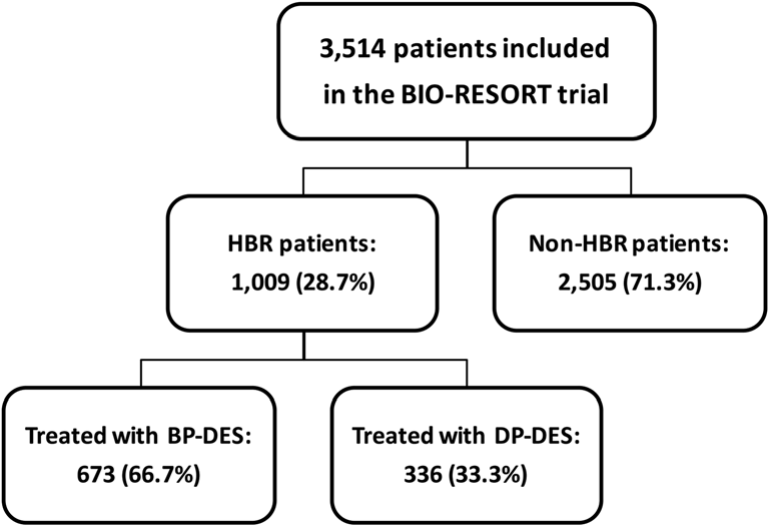
Study flow chart. Abbreviations: BP-DES = biodegradable polymer drug-eluting stent; DP-DES = durable polymer drug-eluting stent; HBR = high bleeding risk

Between December 21, 2012 and August 24, 2015, a total of 3514 patients were included in the BIO-RESORT trial. Of all study participants, 1009 (28.7%) were classified as HBR and 2505 (71.3%) as non-HBR (Fig. 1). Follow-up at 1 year was available in 996 (98.8%) of all HBR patients (1 lost to follow-up, 11 consent withdrawals). Outcome data were used until the time of consent withdrawal.

### High Bleeding Risk Versus Non-high Bleeding Risk Patients

HBR patients were significantly older than non-HBR and had significantly more co-morbidities (Supplementary Table 1). Proportions of all individual HBR criteria are displayed in Table 1. All 1009 HBR patients met a total of 1246 HBR criteria; 798 (79.1%) met 1 HBR criterion, 188 (18.6%) met 2 HBR criteria, and 23 (2.3%) met ≥ 3 HBR criteria. Of all HBR criteria, the most often met criteria were an age ≥ 75 years (50.6%) and the use of oral anticoagulation therapy (30.3%). HBR patients met on average 1.2 HBR criteria. Dual antiplatelet therapy containing aspirin and one of the more potent P2Y_12_ inhibitors (i.e., ticagrelor or prasugrel) was significantly less often prescribed in HBR patients (Supplementary Table 1).

**Table 1:** HBR criteria for all patients and stratified for patients treated with BP-DES versus DP-DES

	HBR population *N* = 1009			
	All HBR patients *N* = 1009	BP-DES *N* = 673	DP-DES *N* = 336	*p* value
Age ≥ 75 years	631 (62.5)	423 (62.9)	208 (61.9)	0.77
Current use of oral anticoagulation	377 (37.4)	260 (38.6)	117 (34.8)	0.24
Hemoglobin < 11 g/dl	91 (9.0)	58 (8.6)	33 (9.8)	0.53
Platelet count < 100,000 mm^3^	11 (1.1)	7 (1.0)	4 (1.2)	0.83
Previous GI-bleeding (within 12 months)	8 (0.8)	4 (0.6)	4 (1.2)	0.31
Previous stroke (within 12 months)	14 (1.4)	9 (1.3)	5 (1.5)	0.85
Previous intracranial hemorrhage (ever)	5 (0.5)	3 (0.4)	2 (0.6)	0.75
Severe renal insufficiency requiring dialysis	10 (1.0)	5 (0.7)	5 (1.5)	0.26
Current use of NSAID	99 (9.8)	58 (8.6)	41 (12.2)	0.07

Values are *n* (%) or mean ± SD

*BP-DES* biodegradable polymer drug-eluting stent, *DP-DES* durable polymer drug-eluting stent, *GI* gastro-intestinal, *HBR* high bleeding risk, *NSAID* non-steroidal anti-inflammatory drug

The Supplementary Table 2 presents the clinical follow-up data for HBR patients versus non-HBR patients. The rate of TVF was significantly higher in HBR patients versus non-HBR patients (6.7 vs. 4.2%, HR 1.59 [95% CI 1.17–2.17], *p* = 0.003). The rates of any death, cardiac death, TLF, MACE, POCE, and major bleeding were also higher in HBR patients (all *p* values ≤ 0.001). There was no statistically significant difference in the rates of MI, target vessel revascularization, target lesion revascularization, and stent thrombosis.

### High Bleeding Risk Patients Stratified for BP-DES Versus DP-DES

Of all 1009 HBR patients, 673 (66.7%) received BP-DES, and 336 (33.3%) received DP-DES. There were no significant between-group differences in baseline characteristics for HBR patients treated with BP-DES versus DP-DES, except for a somewhat higher body mass index in the BP-DES group (27.5 vs. 26.8, *p* = 0.01, Table 2). HBR criteria were similar for both stent groups (Table 1), and there was no statistically significant between-group difference in medication at discharge (Table 2). After 1 year, in both groups, similar proportions of patients were on dual antiplatelet therapy or on oral anticoagulation therapy plus P2Y_12_ inhibitor.

**Table 2:** Baseline characteristics of HBR patients stratified for treatment with BP-DES versus DP-DES

	HBR population *N* = 1009		p log-rank
	BP-DES *N* = 673	DP-DES *N* = 336	
Age (years)	73.7 ± 9.1	73.4 ± 8.9	0.63
Men	414 (61.5)	217 (64.6)	0.34
Body mass index (kg/m^2^)	27.5 ± 4.3	26.8 ± 3.7	0.01
Current smoker	104 (16.1)	65 (20.1)	0.13
Family history of coronary artery disease	230 (36.6)	108 (34.0)	0.43
Diabetes mellitus	159 (23.6)	81 (24.1)	0.87
Hypertension	372 (55.3)	191 (56.8)	0.64
Hypercholesterolemia	259 (38.5)	140 (41.7)	0.33
Previous MI	140 (20.8)	83 (24.7)	0.16
Previous stroke	67 (10.0)	45 (13.4)	0.10
Previous PCI	157 (23.3)	72 (21.4)	0.50
Previous CABG	89 (13.2)	49 (14.6)	0.55
Previous GI-bleeding	19 (2.8)	9 (2.7)	0.90
Clinical presentation			0.06
ST-elevation MI Non-ST-elevation MI Unstable angina Stable angina	185 (27.5) 135 (20.1) 128 (19.0) 225 (33.4)	68 (20.2) 84 (25.0) 70 (20.8) 114 (33.9)	
Acute coronary syndrome	448 (66.6)	222 (66.1)	0.88
Lesion/procedural characteristics			
No. of lesions treated per pt.			0.13
One Two or more	488 (72.5) 185 (27.5)	228 (67.9) 108 (32.1)	
Vascular access site			
Radial Femoral	229 (44.4) 374 (55.6)	159 (47.3) 177 (52.7)	0.38
Treated coronary vessels			
Right coronary artery Left anterior descending artery Circumflex artery Left main Graft	248 (36.8) 308 (45.8) 201 (29.9) 23 (3.4) 25 (3.7)	144 (42.9) 153 (45.5) 95 (28.3) 11 (3.3) 19 (5.7)	0.07 0.95 0.60 0.91 0.16
At least one in-stent restenosis	16 (2.4)	13 (3.9)	0.18
At least one small-vessel*	400 (59.4)	187 (55.7)	0.25
At least one lesion length > 27 mm	203 (30.2)	107 (31.8)	0.59
Discharge medication			
Dual antiplatelet therapy with clopidogrel with ticagrelor/prasugrel	618 (91.8) 402 (59.7) 216 (32.1)	308 (91.7) 209 (62.2) 99 (29.5)	0.93 0.45 0.40
Oral anticoagulation + P2Y_12_ inhibitor	259 (38.5)	115 (34.2)	0.19
Proton pump inhibitor	490 (72.8)	237 (70.5)	0.49
1-year medication	*N* = 644	*N* = 315	
Dual antiplatelet therapy with clopidogrel with ticagrelor/prasugrel	402 (62.4) 231 (35.9) 171 (26.6)	205 (65.1) 126 (40.0) 79 (25.1)	0.30 0.21 0.63
Oral anticoagulation + P2Y_12_ inhibitor	213 (33.1)	92 (29.2)	0.23

Values are *n* (%) or mean ± SD. *All lesions with a reference vessel-diameter of ≤ 2.75 mm were considered to be small-vessels

*CABG* coronary artery bypass graft, *MI* myocardial infarction, *PCI* percutaneous coronary intervention. Other as in Table 1

**Fig. 2:**
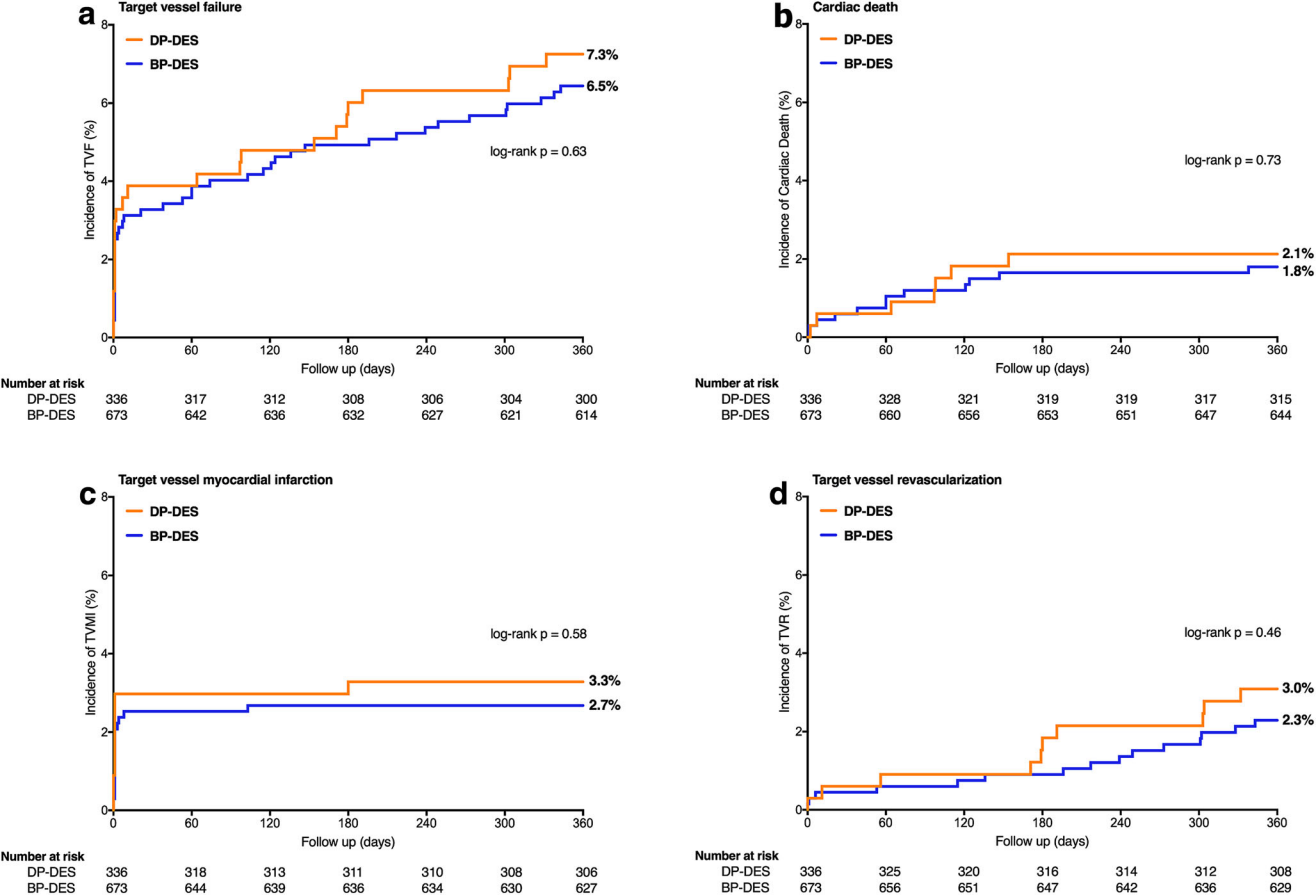
One-year clinical outcomes of HBR patients stratified for treatment with BP-DES versus DP-DES. Kaplan-Meier curves for the primary composite endpoint target vessel failure (**a**) and the individual components thereof: cardiac death (**b**), target vessel myocardial infarction (**c**), and target vessel revascularization (**d**). Abbreviations: BP-DES = biodegradable polymer drug-eluting stent; DP-DES = durable polymer drug-eluting stent; HBR = high bleeding risk

Table 3 presents the clinical outcome data of HBR patients treated with BP-DES versus DP-DES. The primary clinical endpoint TVF was reached in 43/673 (6.5%) HBR patients treated with BP-DES and 24/336 (7.3%) treated with DP-DES (HR 0.88 [95% CI 0.54–1.46], *p* = 0.63, Fig. 2). The results for the primary endpoint were consistent in various subgroups, except for multivessel treatment (Supplementary Fig. 1). Between BP-DES and DP-DES groups, there was also no significant difference in the following secondary endpoints: any death (3.3 vs. 4.8%, *p* = 0.23); cardiac death (1.8 vs. 2.1%, *p* = 0.73); target vessel MI (2.7 vs. 3.3%, *p* = 0.58); TVR (2.3 vs. 3.0%, *p* = 0.46); TLF (6.1 vs. 5.7%, *p* = 0.81); TLR (2.0 vs. 0.9%, *p* = 0.22); MACE (7.6 vs. 8.1%, *p* = 0.77); POCE (9.7 vs. 10.5%, *p* = 0.69); definite or probable stent thrombosis (0.5 vs. 0.6%, *p* = 0.75); and major bleeding (3.3 vs. 3.1%, *p* = 0.84).

**Table 3:** One-year clinical outcome of HBR patients stratified for treatment with BP-DES versus DP-DES

	HBR population *N* = 1009			
	BP-DES *N* = 673	DP-DES *N* = 336	Hazard ratio (95% CI)	p log-rank
Death, any	22 (3.3)	16 (4.8)	0.68 (0.36–1.29)	0.23
Cardiac death	12 (1.8)	7 (2.1)	0.85 (0.33–2.15)	0.73
Myocardial infarction, any	18 (2.7)	11 (3.3)	0.81 (0.38–1.72)	0.58
Target vessel myocardial infarction	18 (2.7)	11 (3.3)	0.81 (0.38–1.72)	0.58
Periprocedural myocardial infarction	14 (2.1)	10 (3.0)	0.70 (0.31–1.57)	0.38
Target vessel revascularization	15 (2.3)	10 (3.0)	0.74 (0.33–1.64)	0.46
Target lesion revascularization	13 (2.0)	3 (0.9)	2.14 (0.61–7.53)	0.22
Target vessel failure*	43 (6.5)	24 (7.3)	0.88 (0.54–1.46)	0.63
Target lesion failure	41 (6.1)	19 (5.7)	1.07 (0.62–1.84)	0.81
Major adverse cardiac events	51 (7.6)	27 (8.1)	0.93 (0.59–1.49)	0.77
Patient-oriented composite endpoint	65 (9.7)	35 (10.5)	0.92 (0.61–1.39)	0.69
Definite stent thrombosis	3 (0.5)	0 (0.0)	–	–
Definite or probable stent thrombosis	3 (0.5)	2 (0.6)	1.34 (0.22–8.02)	0.75
Major bleeding	22 (3.3)	10 (3.1)	1.08 (0.51–2.29)	0.84
Fatal	3 (0.4)	0 (0.0)		
Gastrointestinal	10 (1.5)	5 (1.5)		
Intracranial	3 (0.4)	1 (0.3)		
Other	9 (1.3)	4 (1.2)		

The event rates (expressed as no. and %) were calculated with the use of the Kaplan-Meier method. All target-vessel revascularizations were clinically indicated. *Primary endpoint of cardiac death, target vessel-related myocardial infarction, or clinically indicated target vessel revascularization

Abbreviations: as in Tables 1 and 2

**Fig. 3:**
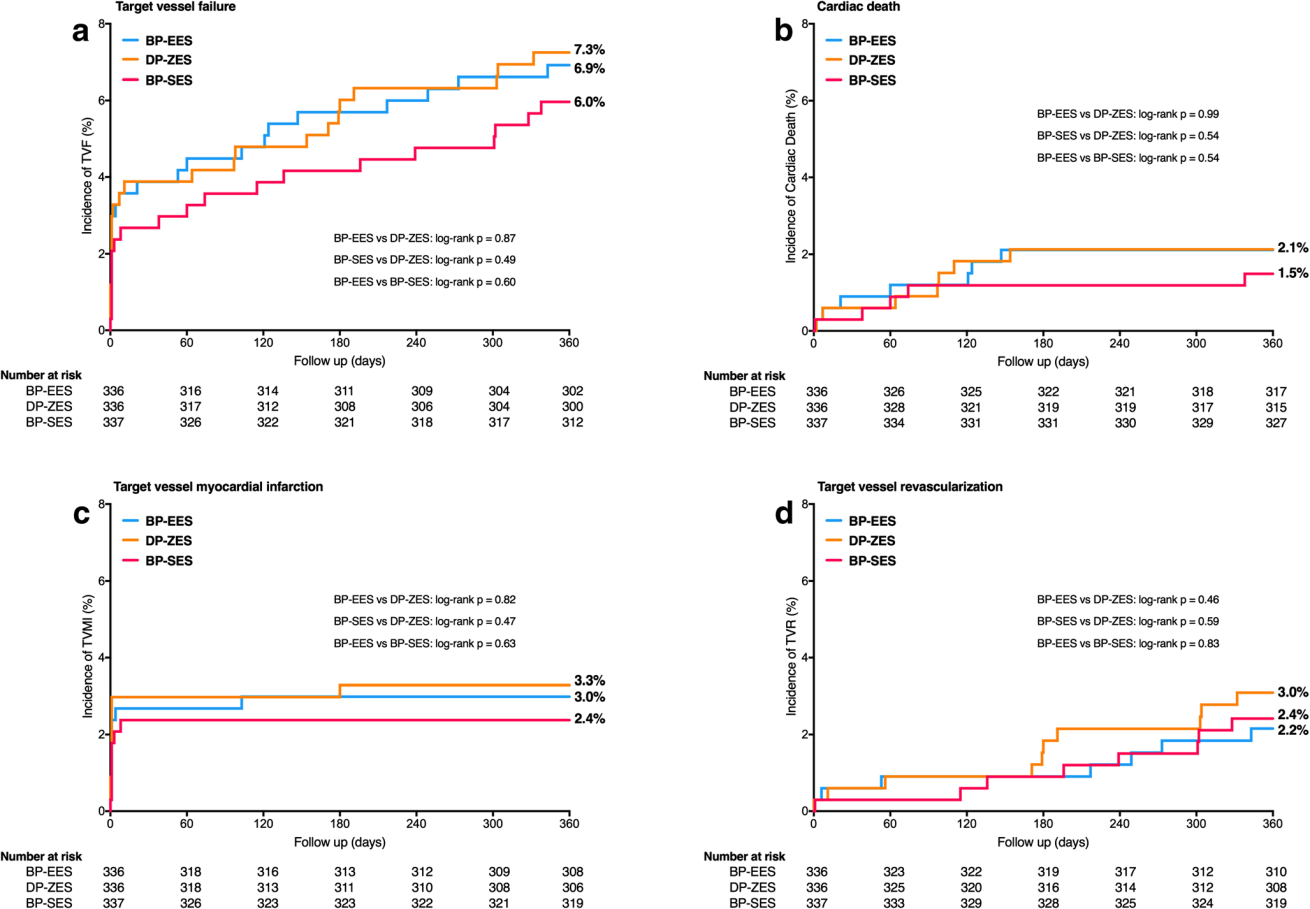
One-year clinical outcomes of HBR patients stratified for treatment with the three individual drug-eluting stents. Kaplan-Meier curves for the primary composite endpoint target vessel failure (**a**) and the individual components thereof: cardiac death (**b**), target vessel myocardial infarction (**c**), and target vessel revascularization (**d**). Abbreviations: BP-EES = biodegradable polymer everolimus-eluting stent; BP-SES = biodegradable polymer sirolimus-eluting stent; DP-ZES = durable polymer zotarolimus-eluting stent; HBR = high bleeding risk

Of all HBR patients in the BP-DES group, 336/673 (49.9%) were treated with Synergy everolimus-eluting stents, and 337/673 (50.1%) were treated with Orsiro sirolimus-eluting stents. Comparison of patients treated with both individual BP-DES versus patients treated with Resolute Integrity DP-DES showed similar results for TVF and all secondary clinical endpoints (Fig. 3, Table 4). In addition, there was no significant difference in clinical outcome between patients treated with Synergy versus Orsiro BP-DES.

**Table 4:** One-year clinical outcome of HBR patients treated with BP-EES, BP-SES, or DP-ZES

	HBR population *N* = 1009			p log-rank		
	BP-EES *N* = 336	DP-ZES *N* = 336	BP-SES *N* = 337	BP-EES vs. DP-ZES	BP-SES vs. DP-ZES	BP-EES vs. BP-SES
Death, any	13 (3.9)	16 (4.8)	9 (2.7)	0.57	0.14	0.36
Cardiac death	7 (2.1)	7 (2.1)	5 (1.5)	0.99	0.54	0.54
Myocardial infarction, any	10 (3.0)	11 (3.3)	8 (2.4)	0.82	0.47	0.63
Target vessel myocardial infarction	10 (3.0)	11 (3.3)	8 (2.4)	0.82	0.47	0.63
Periprocedural myocardial infarction	8 (2.4)	10 (3.0)	6 (1.8)	0.63	0.31	0.59
Target vessel revascularization	7 (2.2)	10 (3.0)	8 (2.4)	0.46	0.59	0.83
Target lesion revascularization	6 (1.8)	3 (0.9)	7 (2.1)	0.32	0.22	0.81
Target vessel failure*	23 (6.9)	24 (7.3)	20 (6.0)	0.87	0.49	0.60
Target lesion failure	22 (6.6)	19 (5.7)	19 (5.7)	0.64	0.95	0.60
Major adverse cardiac events	28 (8.4)	27 (8.1)	23 (6.8)	0.90	0.52	0.44
Patient-oriented composite endpoint	31 (9.3)	35 (10.5)	34 (10.1)	0.61	0.85	0.75
Definite stent thrombosis	2 (0.6)	0 (0.0)	1 (0.3)	0.16	0.32	0.56
Definite or probable stent thrombosis	2 (0.6)	2 (0.6)	1 (0.3)	0.99	0.56	0.56
Any bleeding	14 (4.3)	15 (4.6)	14 (4.2)	0.84	0.80	0.96
Major bleeding Fatal Gastrointestinal Intracranial Other	12 (3.7) 3 (0.9) 5 (1.5) 1 (0.3) 6 (1.8)	10 (3.1) 0 (0.0) 5 (1.5) 1 (0.3) 4 (1.2)	10 (3.0) 0 (0.0) 5 (1.5) 2 (0.6) 3 (0.9)	0.68	0.96	0.64

The event rates (expressed as no. and %) were calculated with the use of the Kaplan-Meier method. All target-vessel revascularizations were clinically indicated. *Primary endpoint of cardiac death, target vessel-related myocardial infarction, or clinically indicated target vessel revascularization

*BP-EES* biodegradable polymer everolimus-eluting stent, *BP-SES* biodegradable polymer sirolimus-eluting stent, *DP-ZES* durable polymer zotarolimus-eluting stent. Other as in Table 1

## Discussion

Almost 30% of the PCI all-comers who participated in the BIO-RESORT trial were at HBR. At 1-year follow-up of this large population of HBR patients, PCI with two very thin-strut BP-DES showed similar safety and efficacy as treatment with a contemporary thin-strut DP-DES. There was no significant between-group difference in the rate of the primary composite endpoint TVF and all prespecified secondary clinical endpoints. Many PCI patients have an increased bleeding risk, but the exact proportion depends on the HBR criteria used and may be higher in patients with acute coronary syndromes. A study that applied similar criteria found a HBR in 42% of patients treated for acute coronary syndromes [9], a rate that matches quite well with the 29% in BIO-RESORT, considering that 30% of the BIO-RESORT all-comers had stable coronary disease. Our results confirm the overall high adverse event risk of HBR patients [11, 15, 20].

So far, no study compared BP-DES and DP-DES specifically in HBR patients. Only two dedicated stent trials assessed HBR patients, comparing polymer-free biolimus A9-coated stent (BioFreedom; Biosensors Europe) [14] or early-generation zotarolimus-eluting DP-DES (Endeavor; Medtronic, USA) [15] with bare metal stents that formerly were used to minimize DAPT duration. The novel stents improved clinical outcome in both trials that applied an abbreviated DAPT regimen of 1 month. Based on these results, guidelines currently recommend DES over bare metal stents in *all* patients [4]. In our HBR patients, event rates were lower than in the drug-coated stent arm of LEADERS FREE [14] for: death (all-cause: 3.8 versus 8.0%; cardiac: 1.4 versus 4.2%); MI (any: 2.9 versus 6.1%); revascularization (TLR: 1.6 versus 5.1%; TVR: 2.1 versus 5.8%); stent thrombosis (definite or probable: 0.5 versus 2.2%); and major bleeding (3.3 versus 7.2%) [14]. These differences may be explained by dissimilarities in: (1) follow-up duration (360 versus 390 days); (2) MI definitions (extended historical ARC versus third universal definition); (3) DAPT duration (almost two thirds of BIO-RESORT HBR patients still on DAPT after 1 year versus 1 month DAPT in LEADERS FREE); (4) strut thickness (uncoated, 60–91 versus 120 μm) which might be relevant as thicker struts increase stent thrombosis, restenosis, and reintervention risk that theoretically could have contributed to the higher bleeding rate in LEADERS FREE; (5) the number of HBR criteria per patient (1.2 versus 1.7) [14]. In addition, in our HBR patients, event rates were lower than in the zotarolimus-eluting DP-DES arm of the ZEUS trial [15] for: death (all-cause: 3.8 versus 15.8%; cardiac: 1.4 versus 11.8%); revascularization (TLR: 1.6 versus 5.2%; TVR: 2.1 versus 5.9%); and stent thrombosis (definite or probable: 0.5 versus 2.6%). These higher event rates may partly be explained by the higher risks of re-stenosis and repeat revascularization associated with the use of Endeavor as compared to newer-generation DP-DES [21, 22], while the very high mortality is related to the old age (80.4 years) in ZEUS participants [15].

Based on the two aforementioned trials [14, 15], the most recent focused update on DAPT of the European Society of Cardiology [23] recommends an abbreviated DAPT duration of 1 to 6 months in HBR. However, it is unclear whether the advantage of such a shortened DAPT regimen in HBR patients may apply to DES other than used in these two trials. Both BioFreedom and Endeavor stents have never been compared to the three DES used in BIO-RESORT. The thin-strut Resolute Integrity DP-DES is a successor of the Endeavor DP-DES and has shown excellent short- and long-term clinical outcomes [5, 24, 25]. The very thin-strut Synergy BP-DES, which in BIO-RESORT was for the first time assessed in all-comers, previously was shown to be non-inferior to durable polymer everolimus-eluting stents in the EVOLVE trials [26, 27]. In addition, Synergy was recently studied in patients ≥ 75 years (i.e., the most common HBR criterion) treated with an abbreviated DAPT regimen in the SENIOR trial [28]. In that trial, patients treated with Synergy had lower rates of TLR and ischemic events as compared to bare metal stents. The safety of the device is currently being studied in HBR patients who are treated with DAPT for only 1 month (POEM study, NCT03112707) or 3 months (EVOLVE short DAPT study, NCT02605447). The very thin-strut sirolimus-eluting Orsiro BP-DES has recently shown favorable results versus everolimus-eluting DP-DES [29, 30]. In addition, in the SORT-OUT IX trial (NCT02623140), Orsiro is being compared to the BioFreedom stent, which was previously tested in LEADERS FREE. Upcoming results of some of the abovementioned trials may reveal the safety of using an abbreviated DAPT regimen in HBR patients treated with a DES, used in the present study.

While the absence of durable polymers in BP-DES may reduce inflammation and promote arterial healing, meta-analyses thus far showed no unequivocal benefit in clinical outcome for broad patient populations treated with BP-DES over DP-DES [5–8]. Our present study adds to the current knowledge that in HBR patients, who have substantially higher event rates than non-HBR patients, there is also no short-term benefit of BP-DES. Nevertheless, the two BP-DES used in our study differ substantially. The polymer of the Synergy BP-DES is completely resorbed in approximately 4 months, while this process takes approximately 18 months in the Orsiro BP-DES [13]. Therefore, potential benefits of the Orsiro may only be seen later than after 1 year. As a result, the mid- and long-term results of BIO-RESORT will be of considerable interest. This is even more the case, as participants in this trial generally followed a stringent policy of DAPT discontinuation after 12 months.

### Limitations

The results of this analysis should be considered hypothesis generating, and the study was not powered to detect differences in rare clinical events such as stent thrombosis. As there is no general HBR definition, we followed criteria applied in previous HBR trials, using data available in the database of the randomized BIO-RESORT trial. In contrast to the previous HBR trials, we had no information of previously diagnosed malignancy and severe liver disease available in our database, and therefore, we might have missed some HBR patients. However, if patients with severe liver disease had reduced levels of hemoglobin or platelet count, they anyway were classified as HBR. Furthermore, we used requirement of dialysis as parameter of severe renal insufficiency rather than a creatinine clearance < 40 cc/min/1.73 m^2^. While this might have lowered the mean number of HBR criteria per patient, many patients with a creatinine clearance below that threshold may have been 75 years or older and, thus, anyway classified as HBR (based on their age). Nevertheless, these minor differences in HBR criteria make it somewhat difficult to compare our results with previous HBR studies. Despite high follow-up rates, and independent monitoring and event adjudication, the event rates were relatively low.

## Conclusion

At 1-year follow-up, very thin-strut BP-DES showed similar safety and efficacy for treating HBR patients as thin-strut DP-DES. Further follow-up assessment of these high-risk patients is required to evaluate the presence or absence of long-term benefits of BP-DES over DP-DES.

## Electronic supplementary material

ESM 1 (DOCX 15 kb)

ESM 2 (DOCX 13 kb)

ESM 3 (DOCX 52 kb)

## Abbreviations

BP-DES: Biodegradable polymer drug-eluting stent (s)
DP-DES: Durable polymer drug-eluting stent (s)
HBR: High bleeding risk
MI: Myocardial infarction
PCI: Percutaneous coronary intervention
TVF: Target vessel failure
