# Supplementary material for: High Bleeding Risk Patients Treated with Very Thin-Strut Biodegradable Polymer or Thin-Strut Durable Polymer Drug-Eluting Stents in the BIO-RESORT Trial
Source: Cardiovasc Drugs Ther. 2018 Aug 24;32(6):567–76. doi: 10.1007/s10557-018-6823-9 (PMC6267643; doi:10.1007/s10557-018-6823-9)
Supplement: Supplementary file 1 — (DOCX 15 kb) [file 10557_2018_6823_MOESM1_ESM.docx]

**Supplementary Table 1. Baseline characteristics stratified for HBR and non-HBR patients.**

|  | **Total population**  **N = 3,514** | |  |
| --- | --- | --- | --- |
|  | **HBR** N = 1,009 | **Non-HBR** N = 2,505 | ***p value*** |
| **Age (yrs.)** | 73.6 ± 9.1 | 60.1 ± 8.8 | <0.001 |
| **Men** | 631 (62.5) | 1,916 (76.5) | <0.001 |
| **Body Mass Index (kg/m^2^)** | 27.3 ± 4.2 | 27.5 ± 4.1 | 0.22 |
| **Current smoker** | 169/969 (17.4) | 862/2,453 (35.1) | <0.001 |
| **Family history of coronary artery disease** | 338/947 (35.7) | 1,219/2,425 (50.3) | <0.001 |
| **Diabetes mellitus** | 240 (23.8) | 384 (15.3) | <0.001 |
| **Hypertension** | 563 (55.8) | 1,061 (42.4) | <0.001 |
| **Hypercholesterolemia** | 399 (39.5) | 936 (37.4) | 0.23 |
| **Previous MI** | 223 (22.1) | 426 (17.0) | <0.001 |
| **Previous stroke** | 112 (11.1) | 119 (4.8) | <0.001 |
| **Previous PCI** | 229 (22.7) | 397 (15.8) | <0.001 |
| **Previous CABG** | 138 (13.7) | 129 (5.1) | <0.001 |
| **Previous GI-bleeding** | 28 (2.8) | 26 (1.0) | <0.001 |
| ***Clinical presentation***  **ST-elevation MI**  **Non-ST-elevation MI**  **Unstable angina**  **Stable angina** | 253 (25.1)  219 (21.7)  198 (19.6)  339 (33.6) | 820 (32.7)  537 (21.4)  422 (16.8)  726 (29.0) | <0.001 |
| **Acute coronary syndrome** | 670 (66.4) | 1,779 (71.0) | <0.01 |
| **Lesion/procedural characteristics** |  | | |
| ***No. of lesions treated per pt.***  **One**  **Two or more** | 716 (71.0)  293 (29.0) | 1,864 (74.4)  641 (25.6) | 0.08 |
| ***Vascular access site***  **Radial**  **Femoral** | 458 (45.4)  551 (54.6) | 1,139 (45.5)  1,366 (54.5) | 0.97 |
| ***Treated coronary vessels***  **Right coronary artery**  **Left anterior descending artery**  **Circumflex artery**  **Left main**  **Graft** | 392 (38.9)  461 (45.7)  296 (29.3)  34 (3.4)  44 (4.4) | 945 (37.7)  1,258 (50.2)  708 (28.3)  42 (1.7)  26 (1.0) | 0.53  0.02  0.52  0.002  <0.001 |
| **At least one in-stent restenosis** | 29 (2.9) | 60 (2.4) | 0.41 |
| **At least one small-vessel*** | 587 (58.2) | 1,491 (59.5) | 0.46 |
| **At least one lesion length > 27mm** | 310 (30.7) | 763 (30.5) | 0.88 |
| ***Randomized Stent***  **Orsiro**  **Resolute Integrity**  **Synergy** | 337 (33.4)  336 (33.3)  336 (33.3) | 832 (33.2)  837 (33.4)  836 (33.4) | 0.99 |
| **Biodegradable polymer stent** | 673 (66.7) | 1,668 (66.6) | 0.95 |
| **Discharge medication** |  |  | |
| **Dual antiplatelet therapy**  **with clopidogrel**  **with ticagrelor/prasugrel** | 926 (91.8)  611 (60.6)  315 (31.2) | 2,493 (99.5)  1,184 (47.3)  1,309 (52.3) | <0.001  <0.001  <0.001 |
| **Oral anticoagulation + P2Y_12_ inhibitor** | 374 (37.1) | - | - |
| **Proton pump inhibitor** | 727 (72.1) | 1285 (51.3) | <0.001 |
| **1-year medication** | N = 959 | N = 2,473 |  |
| **Dual antiplatelet therapy**  **with clopidogrel**  **with ticagrelor/prasugrel** | 607 (63.3)  357 (37.2)  250 (26.1) | 2,332 (94.3)  1,171 (47.4)  1,161 (46.9) | <0.001  <0.001  <0.001 |
| **Oral anticoagulation + P2Y_12_ inhibitor** | 305 (31.8) | 50 (2.0) | <0.001 |

Values are n (%) or mean ± SD. * All lesions with a reference vessel-diameter of ≤2.75 mm were considered to be small-vessels.

Abbreviations: CABG = coronary artery bypass grafting; GI = gastro-intestinal; HBR = high bleeding risk; MI = myocardial infarction; PCI= percutaneous coronary intervention.
