# Supplementary material for: High Bleeding Risk Patients Treated with Very Thin-Strut Biodegradable Polymer or Thin-Strut Durable Polymer Drug-Eluting Stents in the BIO-RESORT Trial
Source: Cardiovasc Drugs Ther. 2018 Aug 24;32(6):567–76. doi: 10.1007/s10557-018-6823-9 (PMC6267643; doi:10.1007/s10557-018-6823-9)
Supplement: Supplementary file 2 — (DOCX 13 kb) [file 10557_2018_6823_MOESM2_ESM.docx]

**Supplementary Table 2. One-year clinical outcome stratified for HBR and non-HBR patients.**

|  | **Total population**  N = 3,514 | |  | |
| --- | --- | --- | --- | --- |
|  | **HBR** N = 1,009 | **Non-HBR** N = 2,505 | **Hazard Ratio**  **(95% CI)** | ***p log-rank*** |
| **Death, any** | 38 (3.8) | 20 (0.8) | 4.82 (2.80-8.28) | <0.001 |
| **Cardiac death** | 19 (1.9) | 11 (0.4) | 4.36 (2.08-9.16) | <0.001 |
| **Myocardial infarction, any** | 29 (2.9) | 56 (2.2) | 1.29 (0.82-2.02) | 0.26 |
| **Target vessel myocardial infarction** | 29 (2.9) | 53 (2.1) | 1.36 (0.87-2.14) | 0.18 |
| **Periprocedural myocardial infarction** | 24 (2.4) | 43 (1.7) | 1.38 (0.84-2.28) | 0.20 |
| **Target vessel revascularization** | 25 (2.5) | 54 (2.1) | 1.18 (0.73-1.89) | 0.51 |
| **Target lesion revascularization** | 16 (1.6) | 36 (1.4) | 1.13 (0.63-2.03) | 0.69 |
| **Target vessel failure*** | 67 (6.7) | 106 (4.2) | 1.59 (1.17-2.17) | 0.003 |
| **Target lesion failure** | 60 (6.0) | 89 (3.6) | 1.70 (1.22-2.36) | 0.001 |
| **Major adverse cardiac events** | 78 (7.8) | 101 (4.0) | 1.95 (1.45-2.62) | <0.001 |
| **Patient-oriented composite endpoint** | 100 (10.0) | 158 (6.3) | 1.60 (1.25-2.06) | <0.001 |
| **Definite stent thrombosis** | 3 (0.3) | 8 (0.3) | 0.94 (0.25-3.55) | 0.93 |
| **Definite or probable stent thrombosis** | 5 (0.5) | 11 (0.4) | 1.14 (0.40-3.29) | 0.81 |
| **Major Bleeding**  **Fatal** | 32 (3.3)  3 (0.3) | 37 (1.5)  1 (<0.1) | 2.27 (1.41-3.66) | 0.001 |

The event rates (expressed as no. and %) were calculated with the use of the Kaplan-Meier method. All target-vessel revascularizations were clinically indicated. * Primary endpoint of cardiac death, target vessel-related myocardial infarction, or clinically indicated target vessel revascularization.

Abbreviations: CI = confidence interval. HBR = high bleeding risk.
