# Supplementary material for: High Bleeding Risk Patients Treated with Very Thin-Strut Biodegradable Polymer or Thin-Strut Durable Polymer Drug-Eluting Stents in the BIO-RESORT Trial
Source: Cardiovasc Drugs Ther. 2018 Aug 24;32(6):567–76. doi: 10.1007/s10557-018-6823-9 (PMC6267643; doi:10.1007/s10557-018-6823-9)
Supplement: Supplementary file 3 — (DOCX 52 kb) [file 10557_2018_6823_MOESM3_ESM.docx]

|  | **BP-DES** | **DP-DES** | **Forest plot** | **Hazard Ratio**  **(95% CI)** | ***p value*** | ***p interaction*** |
| --- | --- | --- | --- | --- | --- | --- |
| All HBR patients | 43/673 (6.5) | 24/336 (7.3) | 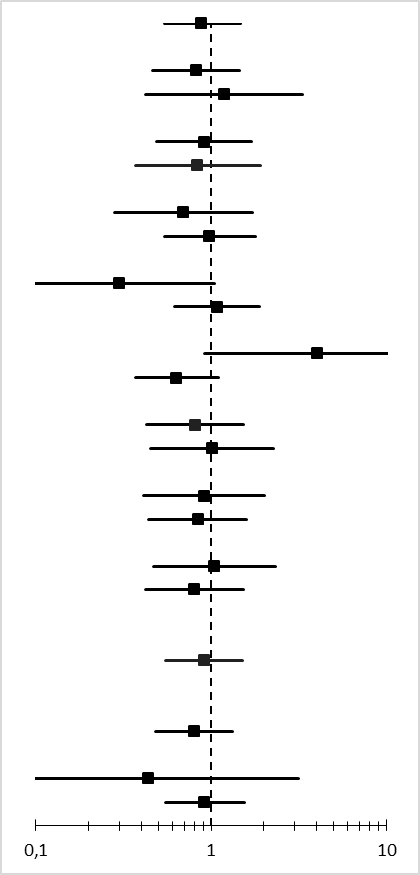 | 0.88 (0.54 – 1.46) | 0.63 |  |
| Men  Women | 30/414 (7.2)  13/259 (5.0) | 19/217 (8.8)  5/119 (4.2) |  | 0.82 (0.46 – 1.45)  1.19 (0.42 – 3.33) | 0.50  0.73 | 0.53 |
| Acute coronary syndrome  Stable angina | 28/448 (6.3)  15/225 (6.7) | 15/222 (6.8)  9/114 (7.9) |  | 0.91 (0.49 – 1.70)  0.84 (0.37 – 1.92) | 0.80  0.68 | 0.88 |
| Diabetes  No diabetes | 11/159 (6.9)  32/514 (6.2) | 8/81 (9.9)  16/255 (6.3) |  | 0.69 (0.28 – 1.71)  0.98 (0.54 – 1.79) | 0.42  0.98 | 0.52 |
| Renal insufficiency  No renal insufficiency | 4/49 (8.2)  39/624 (6.3) | 6/23 (26.1)  18/313 (5.8) |  | 0.30 (0.08 – 1.05)  1.08 (0.62 – 1.89) | 0.07  0.76 | 0.054 |
| Multivessel treatment  Single vessel treatment | 13/124 (10.5)  30/549 (5.5) | 2/75 (2.7)  22/261 (8.4) |  | 4.05 (0.91 – 17.94)  0.63 (0.37 – 1.10) | 0.05  0.11 | 0.02 |
| Small vessel <2.75 mm  No small vessel | 26/400 (6.5)  17/273 (6.2) | 15/187 (8.0)  9/149 (6.0) |  | 0.81 (0.43 – 1.52)  1.01 (0.45 – 2.27) | 0.50  0.94 | 0.66 |
| Bifurcation  No bifurcation | 19/239 (7.9)  24/434 (5.5) | 9/105 (8.6)  15/231 (6.5) |  | 0.91 (0.41 – 2.02)  0.84 (0.44 – 1.60) | 0.85  0.62 | 0.88 |
| Lesion length >27 mm  Lesion length ≤ 27 mm | 18/203 (8.9)  25/470 (5.3) | 9/107 (8.4)  15/229 (6.6) |  | 1.05 (0.47 – 2.33)  0.80 (0.42 – 1.52) | 0.89  0.51 | 0.61 |
| In-stent restenosis  No in-stent restenosis | 0/16 (0)  43/657 (6.5) | 1/13 (7.7)  23/323 (7.1) |  | –  0.91 (0.55 – 1.51) | 0.45  0.74 | 0.88 |
| Bypass graft  No bypass graft | 3/25 (12.0)  40/648 (6.2) | 0/19 (0.0)  24/317 (7.6) |  | –  0.80 (0.48 – 1.33) | 0.25  0.41 | 0.86 |
| Left main  No left main | 2/23 (8.7)  41/650 (6.3) | 2/11 (18.2)  22/325 (6.8) |  | 0.44 (0.06 – 3.13)  0.92 (0.55 – 1.55) | 0.58  0.78 | 0.47 |
|  | | | 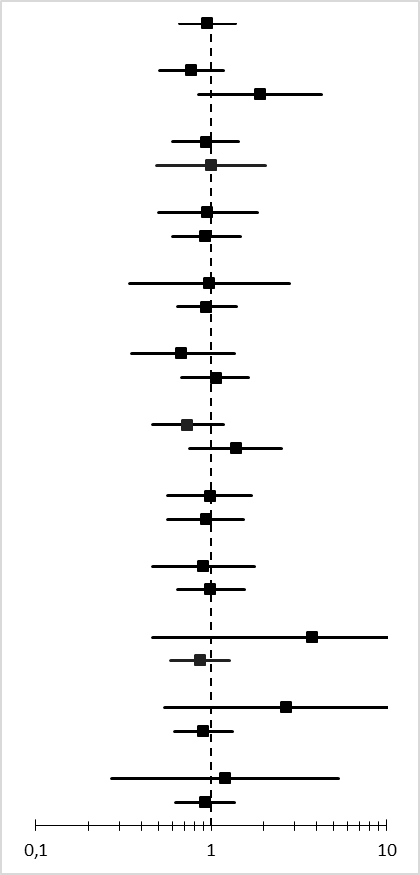 Favors DP-DES EES  Favors BP-DES EES |  | | |

**Supplementary Figure 1. Subgroup analysis of high bleeding risk patients for the primary endpoint target vessel failure.**

Abbreviations: BP-DES = biodegradable polymer drug-eluting stent; DP-DES = durable polymer drug-eluting stent; HBR = high bleeding risk.
